# A prospective randomized controlled pilot study of gut microbiome modulation by *Nigella sativa* seed oil and thymoquinone metabolism in healthy volunteers: implications for gastrointestinal surgery

**DOI:** 10.1038/s41598-026-69567-3

**Published:** 2026-09-15

**Authors:** Ahmad Firas El-Banna, Howard Junca, Arndt Steube, Dirk K. Wissenbach, Utz Settmacher, Claus Schneider, Aysun Tekbaş

**Affiliations:** 1 Department of General, Visceral and Vascular Surgery, Jena University Hospital, Friedrich Schiller University Jena, Am Klinikum 1, 07747 Jena, Germany; 2 Microbial Interactions and Processes (MINP) Research Group, Helmholtz Centre for Infection Research GmbH - HZI, Science Campus Braunschweig-Süd, Inhoffenstrasse 7, 38124 Braunschweig, Germany; 3 RG Microbial Ecology: Metabolism, Genomics & Evolution, Div. Ecogenomics & Holobionts, Microbiomas Foundation, LT11A, 250008 Chia, Colombia; 4 Department of Internal Medicine IV (Gastroenterology, Hepatology, Infectiology, Central Endoscopy), Jena University Hospital, Friedrich Schiller University Jena, Am Klinikum 1, 07747 Jena, Germany; 5 Institute for Forensic Medicine, Jena University Hospital, Friedrich Schiller University, Am Klinikum 1, 07747 Jena, Jena, Germany; 6 Research Programme “Advanced Clinician Scientist Programme”, Interdisciplinary Center of Clinical Research, Medical Faculty of Friedrich Schiller University Jena, Jena University Hospital, Salvador-Allende-Platz 29, 07747 Jena, Germany

**Keywords:** Thymoquinone, Gut microbiome, *Nigella sativa* seed oil, Microbiota modulation, Thymoquinone metabolism, Gastroenterology, Microbiology

## Abstract

Maintaining gut microbial balance may influence outcomes in gastrointestinal surgery. Dietary supplementation has the potential to selectively modify its composition. Nigella sativa seed oil (NSSO), which contains the bioactive compound thymoquinone (TQ), has antimicrobial and immunomodulatory properties, but human data on NSSO-associated gut microbiome changes and urinary TQ detectability after repeated oral exposure remain limited. In this prospective, single-center, randomized open-label pilot study, eight healthy adults were assigned to a treatment group (*n* = 4) receiving TQ-containing NSSO capsules (two capsules, three times daily for 20 days) or a control group (*n* = 4) without supplementation. Fecal samples were collected at five time points and analyzed by 16 S rRNA gene sequencing. Alpha and beta diversity, exploratory differential abundance, and machine learning analyses were performed. Food diaries were reviewed descriptively. Urine samples obtained at two time points were analyzed using Gas Chromatography Mass Spectrometry (GC-MS) to detect TQ and its metabolites. No significant differences in alpha or beta diversity were observed between groups, proving a stable microbiome composition. However, specific taxa increased or decreased, among them *Mediterraneibacter lactaris* (*p* = 9 × 10⁻⁵) and *Terrisporobacter mayombei* (*p* = 3 × 10⁻⁴), respectively. TQ and/or TQ-H₂ were qualitatively detected in urine samples from the treatment group after repeated NSSO intake. This pilot study suggests that repeated NSSO intake is feasible for longitudinal microbiome and urinary metabolite sampling and provides preliminary evidence for taxon-specific changes in healthy adults. Urinary detection of TQ/TQ-H₂ confirms detectability following repeated NSSO intake but does not permit pharmacokinetic conclusions. These findings may guide the design of future blinded, placebo-controlled, diet-controlled perioperative microbiome studies.

Trial Registration: The trial was retrospectively registered at ClinicalTrials.gov (NCT07450807) on 4 March 2026.

**Supplementary Information:**

The online version contains supplementary material available at https://doi.org/10.1038/s41598-026-69567-3.

## Introduction

While the gut microbiome was long regarded merely as a digestive organ, recent studies have revealed that its dense microbial community - comprising bacteria, viruses, fungi, and other microorganisms - forms a complex ecosystem that interacts with the human body and plays key roles in metabolism, immune regulation, and even behavior. One of the most important functions of the gut microbiome is its role in digestion and nutrient absorption, as it breaks down otherwise indigestible food components and contributes to the production of essential nutrients such as vitamins^1^. Moreover, a healthy gut microbiome can support the immune system and provide protection against infections, whereas dysbiosis has been associated with various diseases, including autoimmune disorders and allergic reactions^2,3^. It strengthens the mucosal barrier through gut bacteria-derived short-chain fatty acids (SCFA), such as butyrate, and enhances local intestinal immunity by modulating toll-like receptor expression, antigen-presenting cells, T-cell differentiation, and the formation of lymphoid follicles^4^.

An individual’s microbiome can be described through measures such as diversity, richness, evenness, and stability of its microbial communities^5,6^. Diversity reflects both the number and relative abundance of taxa and can be assessed within a single sample (alpha diversity) or between samples (beta diversity)^6,7^. These samples may originate from different regions of the gastrointestinal tract, from various individuals, or even from distinct populations. A higher microbial diversity is generally associated with greater ecosystem stability, helping the microbiome resist disturbances caused by factors like antibiotics, infection, or immunosuppression^6^. An altered gut microbiome composition has been linked to symptoms such as abdominal pain and bloating, as well as to conditions including inflammatory bowel disease (IBD), nonalcoholic steatohepatitis, and irritable bowel syndrome^6,8^. It was shown that patients with IBD tend to have less bacterial diversity and lower numbers of certain bacteria which together contribute to lower levels of butyrate^6^. Furthermore, gastrointestinal surgery and perioperative factors such as fasting, bowel preparation and antibiotic use disrupt the epithelial barrier and thereby significantly affect the gut microbiome^6^. For instance, butyrate - commonly associated with anti-inflammatory effects in the gut^4,9^- has been shown to increase anastomotic strength in end-to-end intestinal anastomoses in rats^10^. This might have clinical relevance, as intestinal anastomoses carry an average leakage risk of 10–15% despite advanced surgical techniques and perioperative care^10,11^, suggesting that the composition of the gut microbiome could play an important role in postoperative complications. Although it is well established that surgery alters the gut flora, the specific pre- and postoperative microbial dynamics are still not well characterized. Most existing studies have a narrow focus - often addressing bariatric surgery, treatment outcomes, or animal models - leaving the clinical implications for surgical healing largely unexplored^6^.

Given its multifaceted roles, maintaining and understanding the gut microbiome is of paramount importance. Beyond surgical implications, the composition of the gut microbiome can also be rapidly modulated through dietary interventions^9,12^. This offers potential avenues for preventive and therapeutic modulation through dietary supplements. The growing consumption of dietary supplements is indicative of broader societal transformations, including demographic aging, an accelerated lifestyle, and evolving work and social structures^13^. In the presence of existing illnesses, the use of dietary supplements without adverse effects often enhances treatment adherence and improves quality of life^14^. *Nigella sativa* (NS) is a medicinal plant widely used in traditional medical systems such as Unani-Tibb, Ayurveda, Chinese medicine, Siddha, and Prophetic medicine. It is native to Southern Europe, North Africa, and Southwest Asia. Thymoquinone (TQ), the main constituent of its essential oil, is considered the primary bioactive compound responsible for its various effects, including hepatoprotective, anti-inflammatory, antifibrotic, anti-/proapoptotic, anticarcinogenic and antioxidative properties^15^. The side effect profile of TQ is very low, and the LD50 value for oral administration is 2,400 mg/kg^16^. Not much is known so far about the metabolism of TQ. Studies suggest that TQ is transformed into less toxic metabolites in the gastrointestinal tract or into Dihydrothymoquinone in the liver which then undergoes further oxidation and hydroxylation to form Thymoquinol (TQ-H2)^15,17^. Primarily, cytochrome P450 enzymes are involved in the metabolism. Metabolites of TQ are excreted from the body through both urine and feces^18^. In a previous study, we were unable to detect them in serum or urine using untargeted Gas Chromatography Mass Spectrometry (GC-MS) and Liquid Chromatography Mass Spectrometry (LC-MS), possibly due to compound instability, unknown metabolic pathways, or the use of a single oral dose^19,20^. Few animal studies have described the effects of *Nigella sativa* seed oil (NSSO) and its polysaccharide fraction (NSSP) on the gut microbiome. Akinrinde et al. (2022) demonstrated that NSSO protects against cadmium-induced intestinal toxicity in rats by preserving the mucus layer, microbial balance, and immune-inflammatory function^21^. Liang et al. (2021) showed that NSSP modulates the gut microbiota and enhances its diversity, thereby exerting immunomodulatory and metabolic effects. Metagenomic and proteomic analyses revealed that NSSP influences lipid metabolism, polysaccharide biosynthesis, signal transduction, and SCFA production^22^. In contrast, Kumar et al. (2017) found that NS seeds had no major effects on intestinal morphology or overall bacterial counts in broiler chickens, except for a dose-dependent reduction in *Salmonella* spp^23^.

Given the limited human data on NSSO-associated microbiome changes and TQ detectability after repeated oral exposure, the primary aim of this study was to assess whether repeated intake of TQ-containing NSSO capsules is associated with detectable changes in fecal microbial composition in healthy adults. A second aim was to evaluate whether TQ and/or TQ-H₂ can be detected in urine after repeated exposure using untargeted GC-MS. The study was designed as an exploratory proof-of-concept pilot study to generate preliminary human data that may inform the design of future adequately powered, blinded, placebo-controlled studies in perioperative microbiome research.

## Materials & methods

The experimental protocol was approved by the Ethics committee of the Friedrich Schiller University Jena (Registration No. 2024-3273-BO). Written informed consent was obtained from all participants prior to their inclusion in the study.

### Study design (Fig. 1)

**Fig. 1:**
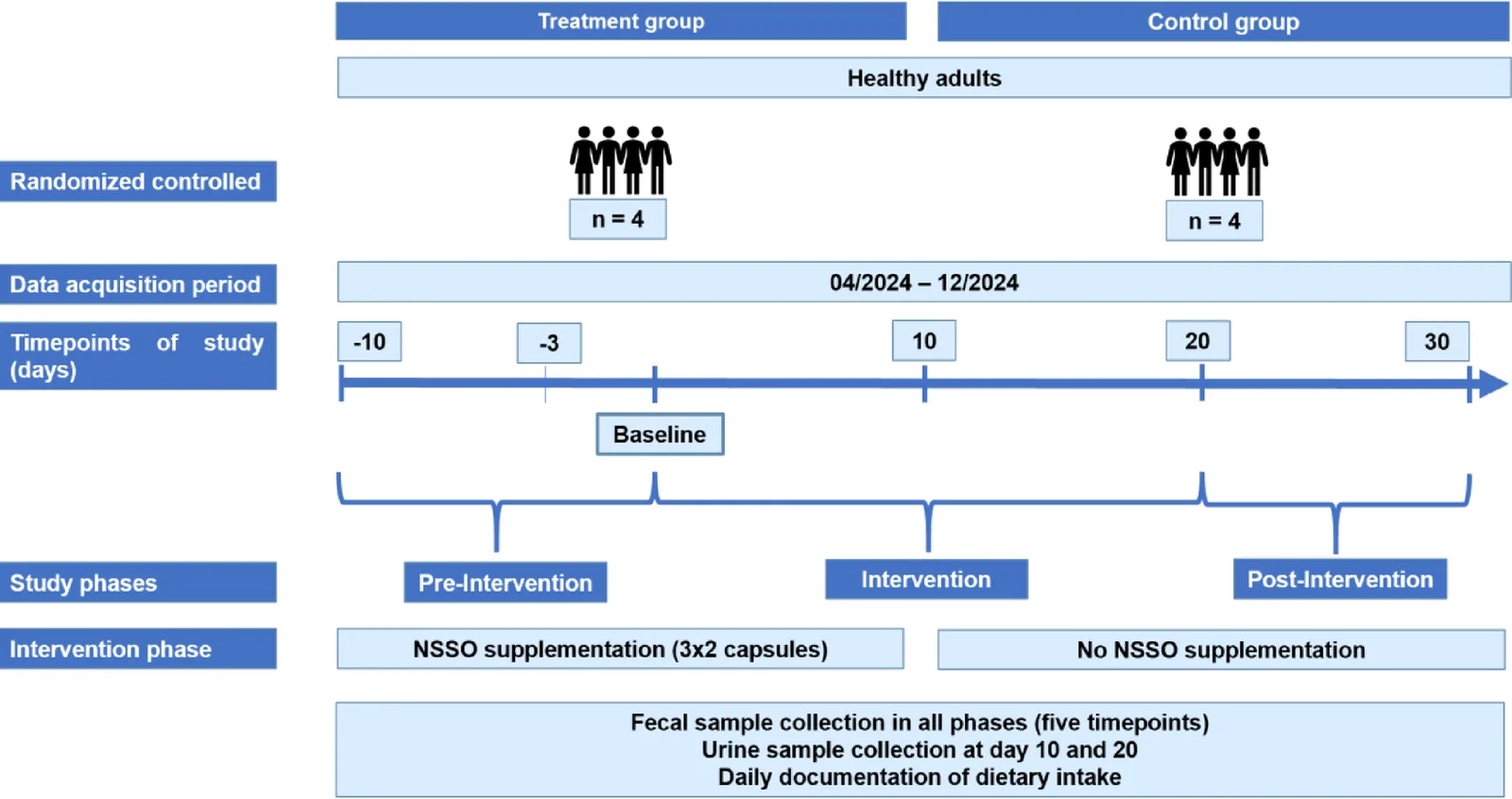
Study design for exploratory assessment of fecal microbial composition and urinary TQ/TQ-H₂ detectability in healthy participants receiving TQ-containing NSSO capsules or no supplementation.

This prospective, single-center, randomized controlled pilot study was conducted on eight healthy adult participants aged 18 years and older at the Jena University Hospital (JUH), Germany. Inclusion criteria required participants to have no recent medication use, no history of gastrointestinal surgery, no pregnancy, and no allergies to NSSO or its derivatives. Participants were excluded if any of these conditions were present. Randomization was performed using a computer-generated random sequence (block size = 4), allocating participants into two groups: a treatment (trtm) and a control group (ctrl). Participant enrolment and allocation to the intervention or control group were performed by the study investigators. This proof-of-concept pilot study followed an open-label design; neither participants nor investigators were blinded to group allocation, and no placebo was used. No formal sample size calculation was performed, as no prior human data were available to estimate the expected effect size of repeated NSSO intake on the gut microbiome or urinary TQ/TQ-H₂ detectability.

Participants in the treatment group received two capsules of NS oil three times daily for 20 days, whereas those in the control group received no supplementation and served as a comparator to distinguish intervention-related changes from the natural temporal variation of the gut microbiome during the study period. The 20-day intervention period was selected pragmatically and was informed by previous preclinical studies evaluating NS, NSSO, or related preparations in microbiome-associated experimental settings^21–23^. Fecal samples were collected from all participants at five distinct time points: 10 and 3 days prior to baseline, and at 10, 20, and 30 days post-baseline. To ensure standardized and aseptic sample collection, all participants were provided with a dedicated stool collection kit. Urine samples were collected at day 10 and 20 from baseline. Each participant maintained a food diary. Food diaries were reviewed descriptively as a taxon-oriented plausibility assessment of dietary confounding. For bacterial taxa identified in the exploratory microbiome analyses, reported intake patterns were screened for food groups with potential relevance to these taxa and to gut microbiome composition. The assessment included soy products, fiber-rich and prebiotic foods, plant-based food intake, fermented foods such as yoghurt or sauerkraut, lactose-containing products, fruit intake, refined grain products, animal protein, fish, omega-3-rich foods, saturated and unsaturated fatty acids, and selected dairy-fat sources such as butter and cheese. Intake tendencies were compared descriptively between the treatment and control groups and categorized as low, balanced, or frequent. No formal dietary scoring system or multivariable dietary adjustment was applied because of the small sample size and the exploratory design. Samples were immediately stored at -20 °C following collection and maintained under these conditions until all specimens had been obtained. Upon completion of the collection phase, all fecal samples were transported to the analytical laboratory for 16 S rRNA gene amplicon sequencing under controlled conditions, utilizing dry ice to preserve sample integrity and prevent microbial degradation. The urine samples were analyzed at the ISO 15,189 certified diagnostic laboratory of the JUH.

### *Nigella sativa* seed oil capsules

NSSO capsules from Tasnim© were selected for their comparatively high TQ content (24.4 g/L TQ, 12.195 mg per capsule), making them well-suited for clinical studies^20^.

### Microbiome analysis

Sample processing and DNA extraction: Fecal samples were processed in a blinded manner with respect to group allocation using the ZymoBIOMICS^®^ Targeted Sequencing Service (Zymo Research Europe GmbH, Freiburg im Breisgau, Germany). DNA was extracted using the automated ZymoBIOMICS^®^-96 MagBead DNA Kit, an automated system to ensure high-quality DNA yield.

Library preparation: Bacterial profiling was performed by amplification of the V1–V2 hypervariable regions of the 16 S rRNA gene using the Quick-16 S™ NGS Library Prep Kit. PCR conditions were optimized to minimize amplification errors, and positive and negative controls were included to monitor assay performance and contamination. Sequencing libraries were generated from the resulting amplicons and sequenced at Zymo Research. Raw sequencing data were used to assess bacterial composition, diversity, and relative abundances before and after NS oil supplementation.

Sequencing and data analysis: Sequencing was performed on an Illumina^®^ NextSeq™ platform, with PhiX added to enhance sequencing quality. Raw 16 S rRNA gene amplicon reads were processed using DADA2 software package (v1.26) (https://benjjneb.github.io/dada2/) following the developer’s recommended pipeline (https://benjjneb.github.io/dada2/tutorial.html) with the following paired-end filtering parameters: out <- filterAndTrim(fnFs, filtFs, fnRs, filtRs, compress = TRUE, truncQ = 2, truncLen = c(232,220), trimLeft = c(1,1), maxN = 0, maxEE = c(2,2), rm.phix = TRUE, matchIDs = TRUE, multithread = TRUE)^24^. After quality filtering, chimera removal, inference of amplicon sequence variants (ASVs), and generation of the ASV count table, relative abundances and taxonomic assignments were determined. Taxonomic classification was performed using the RDP Naïve Bayesian classifier implemented in DADA2 together with Ribosomal Database Project (RDP) trainset No. 19 (https://zenodo.org/records/14168771)^25^. ASVs that remained unclassified at the species level (< 50% bootstrap confidence) were further curated by comparison with the LPSN, EzBiocloud, and NCBI 16 S databases whenever improved taxonomic resolution could be achieved.

Statistical analyses: Statistical analyses and visualizations, including α-diversity indices (Shannon, Chao1, Simpson), β-diversity (Bray-Curtis dissimilarity; PERMANOVA), principal coordinates analysis (PCoA), non-metric multidimensional scaling (NMDS), differential abundance analysis (MaAsLin2), LEfSe, and Random Forest, were performed in MicrobiomeAnalyst 2.0 (https://www.microbiomeanalyst.ca/)^26^ using the parameters implemented in version 2.0 as described by Lu et al. 2023^27^ and available at https://github.com/xia-lab/MicrobiomeAnalystR. Differential abundance analysis was conducted with group/timing status as the primary metadata variable and participant ID as a blocking factor to account for repeated sampling. The main differential-abundance comparison was performed between post-baseline control samples and post-baseline treatment samples (Ctrl_after vs. Trtm_after). For beta-diversity analyses, PERMANOVA permutations were constrained by participant ID. Multiple hypothesis testing was corrected using the Benjamini-Hochberg false discovery rate (FDR), and taxa with an FDR-adjusted p-value (q-value) < 0.05 were considered statistically significant. LEfSe, Random Forest, and simple between-group comparisons were used as exploratory feature-identification approaches and interpreted in conjunction with the MaAsLin2 results rather than as standalone confirmatory analyses. Reported differential-abundance findings were considered exploratory and used to identify candidate taxa for future studies rather than to confirm definitive treatment effects. Dietary variables were not included in multivariable models because of the limited sample size but were evaluated descriptively as potential confounders.

### GC-MS analysis of urine samples

Urine specimens were thawed, and 500 µL were extracted under basic conditions using liquid-liquid extraction with a mixture of ethyl acetate, 2-propanol, and dichloromethane (60:20:20, v/v/v). After evaporation, the extracts were derivatized by acetylation using a mixture of acetic anhydride and pyridine (3:2, v/v). Untargeted GC-MS analysis was performed on a Shimadzu QP 2010 Ultra GC-MS system (Shimadzu, Kyoto, Japan) using a VF-1ms capillary column (30 m × 0.25 mm × 0.25 μm; Restek, Bad Homburg, Germany) with helium (> 99.99%) as the carrier gas. The analysis was completed within 15 min by gradient elution. The MS operated in full-scan mode (50–500 Da). The interface temperature was set to 280 °C, while the MS EI source was maintained at 200 °C. The injection volume from the capped vial to the GC-MS column was 1 µL. Components were identified by matching with the NIST EI library database.

## Results

### Demographic data

Baseline characteristics are summarized in Table 1. Eight participants were randomized equally to the treatment (*n* = 4) and control (*n* = 4) groups. No participant was lost to follow-up or excluded after randomization. None of the participants had a relevant pre-existing medical condition requiring ongoing medication See table 2.

**Table 1:** Baseline characteristics of the study participants. Date are presented as mean (SD) or n (%).

Characteristic	Treatment group (*n* = 4)	Control group (*n* = 4)
Age, years	25.8 (3.1)	24.5 (3.4)
Height, m	1.75 (0.07)	1.79 (0.10)
Weight, kg	71.0 (8.0)	75.0 (12.1)
BMI, kg/m²	23.4 (2.7)	23.4 (1.7)
Sex		
Female	1 (25%)	2 (50%)
Male	3 (75%)	2 (50%)
BMI category		
Underweight	0 (0%)	0 (0%)
Normal weight	2 (50%)	3 (75%)
Overweight	2 (50%)	1 (25%)

**Table 2:** Significantly altered bacterial taxa after NSSO supplementation and their potential functional implications (sorted by phylum).

Phylum	Species	Outcome	Effect / Functional relevance
Firmicutes	*Romboutsia timonensis*	↓ Decrease	Anaerobic carbohydrate and amino acid fermenter; decreases gut fermentation and energy metabolism^37,38^
	*Anaerobutyricum hallii*	↓ Decrease	Butyrate and SCFA producer; lowers anti-inflammatory capacity and epithelial energy support^39,40^
	*Intestinibacter bartlettii*	↓ Decrease	Linked to immune-related adverse events and gallate metabolism; reduces pro-inflammatory potential^34–36^
	*Terrisporobacter mayombei*	↓ Decrease	Acetate-utilizing fermenter; beneficial (linked to ovarian insufficiency risk)^32,33^
	*Turicibacter bilis*	↓ Decrease	Associated with bile acid metabolism; alters bile–microbe interaction^41^, overgrowth may be harmful^6,42^
	*Mediterraneibacter lactaris*	↑ Increase	Carbohydrate-metabolizing; enhances anti-inflammatory effects^31^
	*Dysosmobacter welbionis*	↑ Increase	Butyrate producer linked to improved metabolic and colonic health^29^
	*Blautia schinkii*	↑ Increase	Acetogenic fermenter of carbohydrates; balances SCFA levels and metabolic activity^30^
Actinobacteria	*Bifidobacterium longum*	↓ Decrease	Probiotic species with anti-inflammatory and mucosal protective functions; reduces gut resilience^33,43–46^
	*Bifidobacterium adolescentis*	↓ Decrease	Reduces anti-inflammatory and hepatoprotective roles^33,43–46^
	*Collinsella bouchesdurhonensis*	↓ Decrease	Enriched in sickle cell anemia and type 2 diabetes; metabolic role unclear^47,48^
Bacteroidota	*Pseudaquidulcibacter saccharophilus*	↓ Decrease	Saccharolytic capacity indicated by starch hydrolysis and acid production from multiple carbohydrates; role in human gut unknown^49^
	*Phocaeicola vulgatus*	↑ Increase	Fiber-degrading SCFA producer, enhances metabolic and anti-inflammatory capacity^28,50^

### Sample identification and representation

Each participant contributed five fecal samples. A unique code representing the respective samples of each participant was assigned to the 40 samples. The codes are displayed in the graphs where necessary; however, to improve readability, colors and group labels (red = ctrl, blue = trtm) are primarily used. A complete list of sample identifiers is provided in (Supplementary Digital Content (SDC) 1).

### Fecal microbial composition of treatment and control patients

**Fig. 2:**
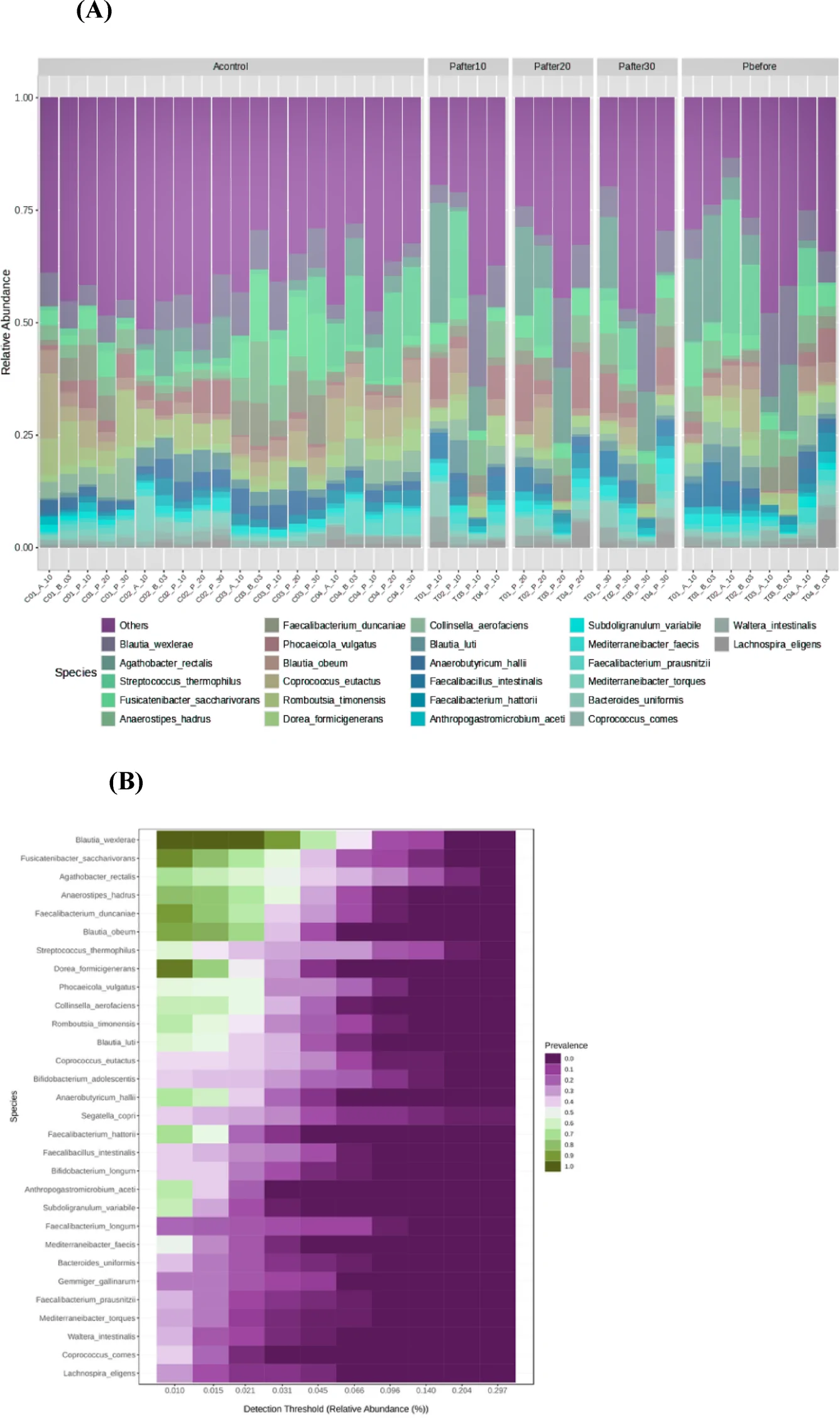
Overall taxonomic composition of the study samples at species level. (**A**) Normalized frequencies of the top 25 bacterial species found in all samples from all control patient samples and patients before and after NSSO administration. (**B**) Heatmap showing the core microbiome of bacterial species and abundance across all the patient samples.

The 16 S amplicon data obtained from fecal samples of both groups demonstrate near-complete coverage, indicating that the sequencing process reached saturation in all cases (SDC 2). This ensures that the data from the samples are comparable, as no significant sequencing depth was lacking during data acquisition. The core microbiome heatmap shows that the overall bacterial composition of the fecal material in all patients from both groups is similar, featuring bacterial types typically found in healthy individuals (Fig. 2). The predominant genera identified in this study include *Blautia*,* Fusicatenbacter*,* Agathobacter*,* Faecalibacterium*,* Anaerostipes*,* Bacteroides*,* Bifidobacterium*,* Streptococcus*,* Coprococcus*,* Romboutsia*,* Subdoligranulum*,* Collinsella*,* Roseburia*,* Ruminococcus*, and *Dorea*, which belong to the most common and abundant taxa in the human gut microbiome.

### Diversity metrics analysis

**Fig. 3:**
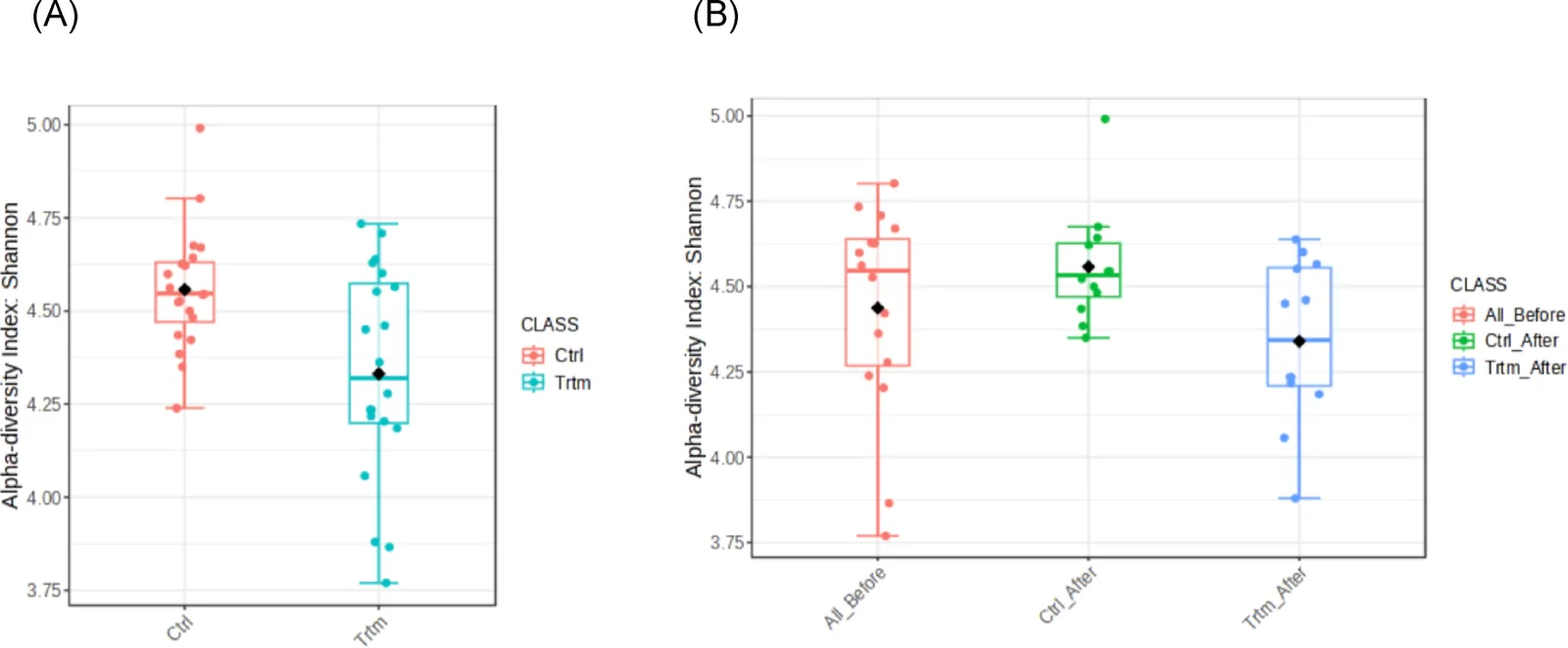
Microbiome Shannon diversity of ASVs between (**A**) each group (control and treatment showed a significant source of variation in the dataset (p-value: 0.021077; [Mann-Whitney] statistic: 285)), while (**B**) the administered treatment as a whole has not an strong effect on gut microbiome Shannon alpha diversity (p-value: 0.16995; [Kruskal-Wallis] statistic: 3.5445).

**Fig. 4:**
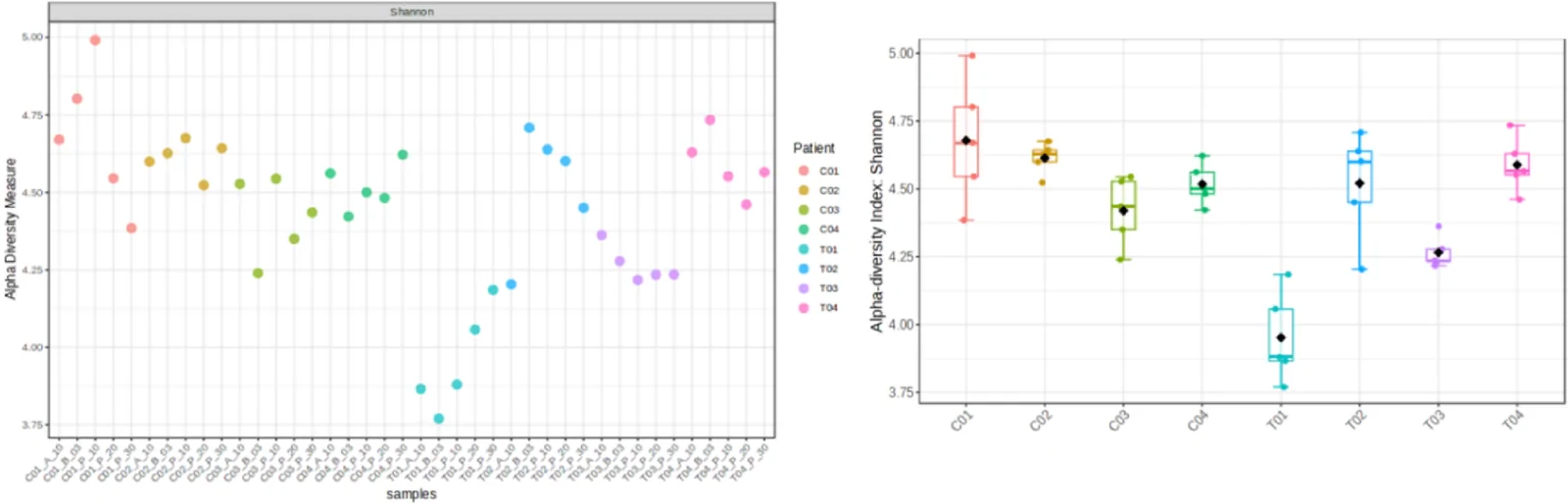
At ASV level, there is a statistically significant difference of the eight individuals regarding their microbiome compositions, indicating a characteristic diversity per person across time. This is the predominant source of similarity/variation between samples (p-value: 0.00057169; [Kruskal-Wallis] statistic: 25.692). Trtm group: C01-C04; Ctrl group: T01-T04.

**Fig. 5:**
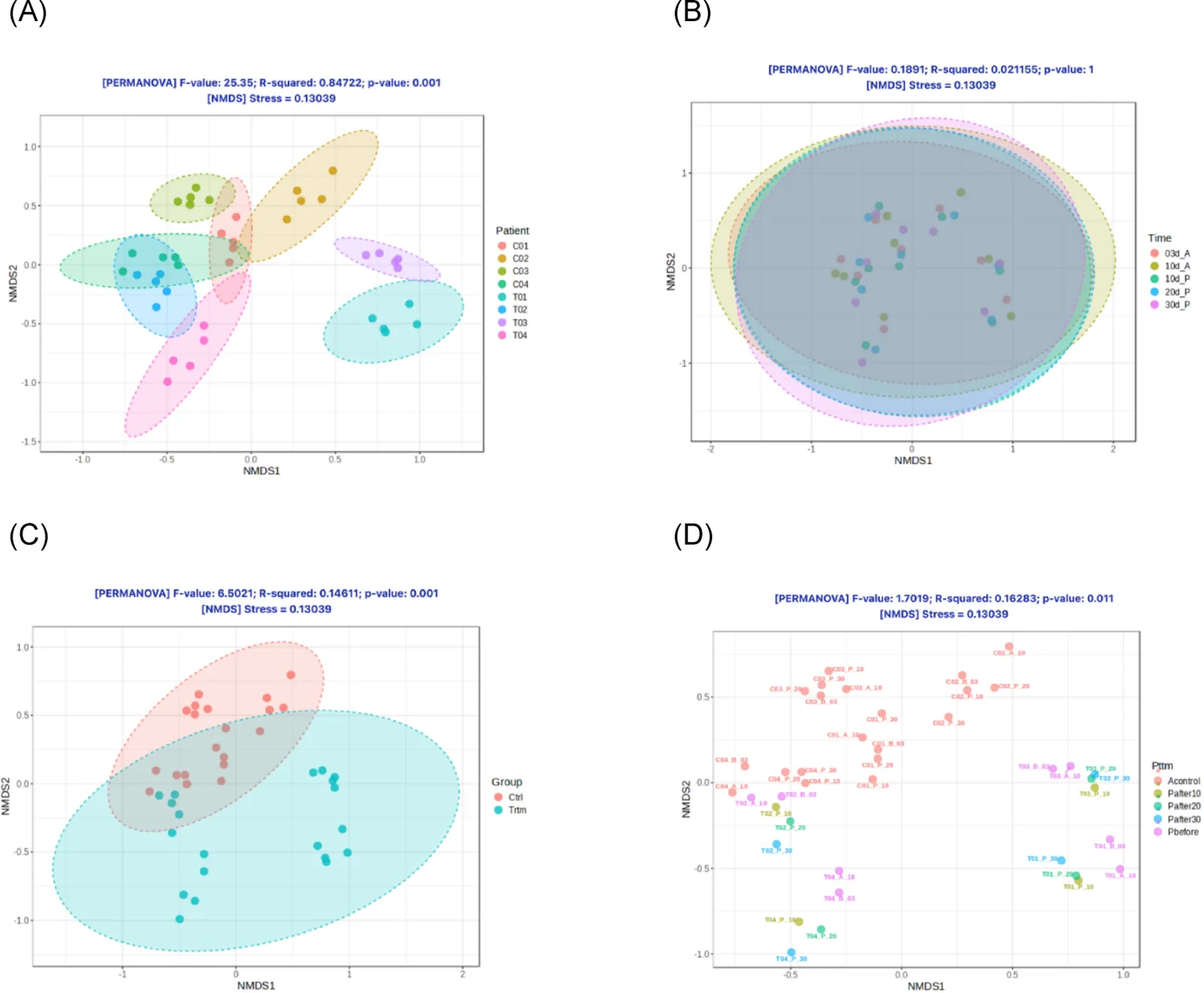
Beta diversity at ASV level of samples from control patients and under NSSO administration. (**A**) Highly significant beta diversity (Nonmetric Multidimensional Scaling (NMDS)) microbiome association within patient samples (Bray-Curtis distance, at ASV level, pairwise PERMANOVA) (**B**) No significant association with time is evidenced across all samples. (**C**) Differences between the control and treatment groups are observed (**D**) Dispersion after disaggregating the samples from control group (Acontrol), the samples of the treatment group before (Pbefore), 10, 20, and 30 days after the beginning of the administration (Pafter). The detected changes in microbiome composition between groups could be attributed to contribution of samples from patients under treatment.

No significant differences were detected when comparing trtm and ctrl group combined before baseline, trtm and ctrl group separately after baseline (Fig. 3). This shows that the specific microbiome diversity of each individual is the predominant source of variation in the dataset, while the administered treatment has no substantial impact on the gut microbiome alpha diversity (*p*-value 0.12). Although the participants in both groups exhibited shared prevalence of several genera, the analysis at the ASV resolution revealed that each participant possessed a distinct and relatively stable microbiome diversity profile. This profile was found to be statistically different from those of the other participants (*p*-value 5 × 10^− 4^), suggesting a specific diversity pattern that remains consistent across time and treatment for each patient (Fig. 4). Beta diversity analysis using the Bray-Curtis dissimilarity matrix (Fig. 5) confirmed these findings as no significant association with time was observed, with clustering by individual group being the main driver of similarity. Overall, no substantial compositional changes were noted between control and treated participants. Each individual’s microbiome displayed distinct intraspecific variation (40%) across time points, with no major shifts in treatment or control groups.

### Identification of differentially abundant bacterial types

**Fig. 6:**
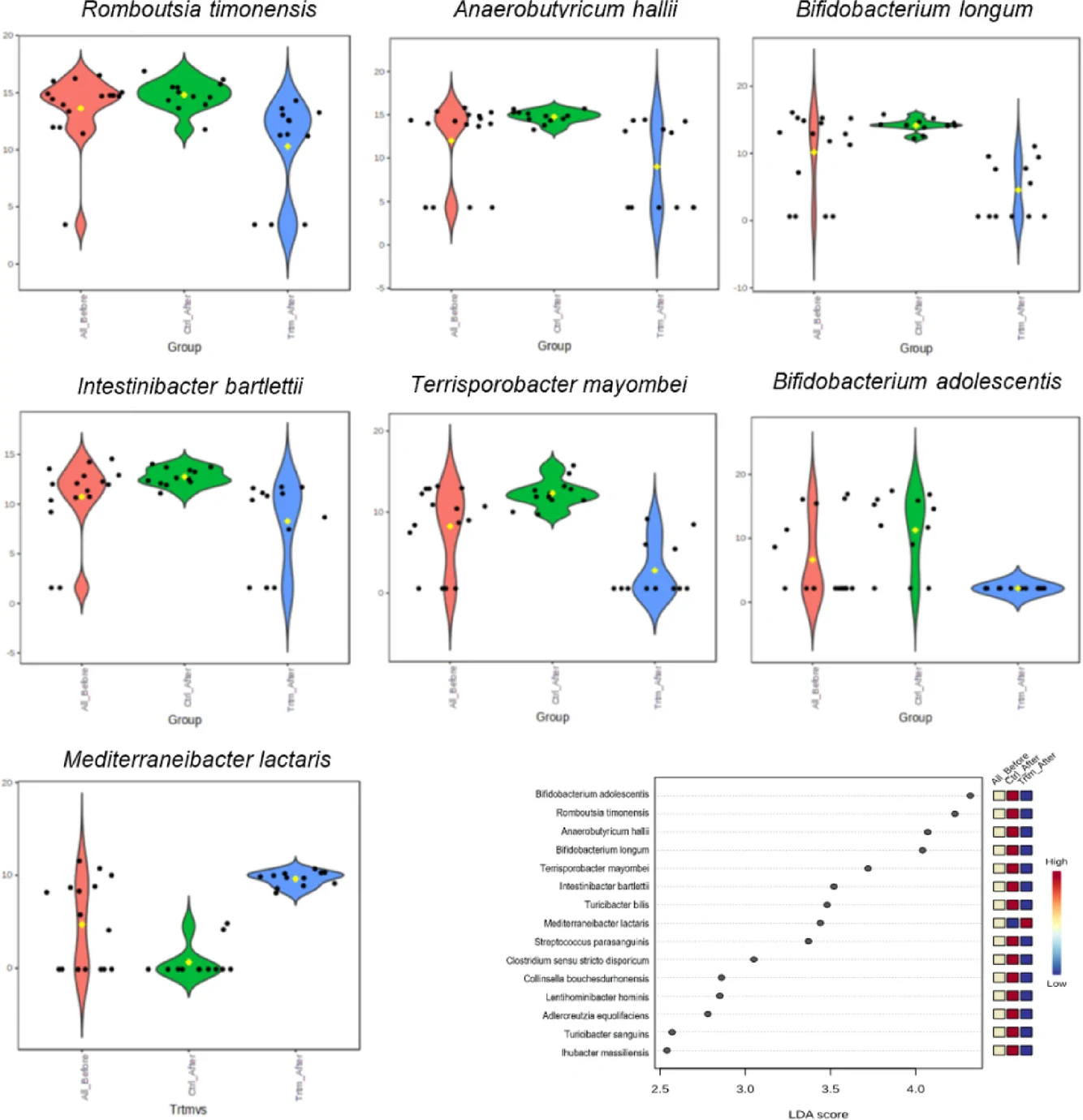
LEfSe analysis showing significantly different bacterial taxa between control, pre-treatment, and post-treatment samples (*p* < 0.05). Because LEfSe does not account for the repeated-measures structure of the dataset, the identified taxa were cross-checked using MaAsLin2 with participant ID included as a blocking factor and Benjamini–Hochberg FDR correction. The significant taxa identified by LEfSe were confirmed by MaAsLin2, although LEfSe may slightly overestimate statistical significance in longitudinal datasets. Mediterraneibacter lactaris showed the strongest increase following NSSO treatment (*p* = 9 × 10⁻⁵). The lower right panel shows the corresponding LDA scores for taxa contributing to group separation.

Differential-abundance analyses indicated selective changes in individual bacterial taxa rather than broad alterations of the overall microbiome composition. Using MaAsLin2, which accounted for the repeated-measures design by including participant ID as a blocking factor and applying Benjamini–Hochberg false discovery rate correction, *Mediterraneibacter lactaris* was significantly enriched following treatment (*p* = 9 × 10⁻⁵). In contrast, *Romboutsia timonensis* (*p* = 0.001), *Bifidobacterium longum* (*p* = 5 × 10⁻⁴), *Terrisporobacter mayombei* (*p* = 3 × 10⁻⁴), *Pseudaquidulcibacter saccharophilus* (*p* = 0.003), and *Lentihominibacter hominis* (*p* = 0.003) were significantly reduced (Fig. 6). Exploratory LEfSe and Random Forest analyses identified largely overlapping taxa (SDC 3), supporting the consistency of these signals, although these findings should be interpreted as exploratory because repeated measures were not accounted for.

### Dietary habits of the participants

Food diaries were evaluated descriptively to explore dietary patterns with potential relevance to the gut microbiome. Descriptive comparisons suggested more frequent fiber and fermented food intake in the control group, whereas plant-based foods and lactose-containing products were reported more frequently in the treatment group. Omega-3 consumption was low in both groups, and animal protein was preferred in both groups. Residual dietary confounding cannot be excluded.

### Thymoquinone detection in urine samples

TQ and/or its metabolite TQ-H₂ were qualitatively detected in urine samples from the treatment group after repeated NSSO intake. No additional TQ-related metabolites were identified using the described untargeted GC-MS approach. Because the method was not validated for absolute quantification, the findings are reported as urinary detectability rather than metabolite concentrations. No adverse events were reported during the study.

## Discussion

This exploratory pilot study showed that repeated intake of TQ-containing NSSO capsules was associated with selective changes in individual gut microbial taxa, while no broad alterations in alpha or beta diversity were observed. In addition, TQ/TQ-H₂ was detectable in urine following repeated oral intake. These findings suggest preservation of overall gut microbiome stability despite selective taxonomic modulation. Among the taxa affected, *Phocaeicola vulgatus* - a fiber-degrading SCFA producer with anti-inflammatory and metabolic benefits - increased in abundance^28^. *Dysosmobacter welbionis* (a butyrate producer linked to metabolic and colonic health)^29^, *Blautia schinkii* (an acetogenic carbohydrate fermenter)^30^, and *Mediterraneibacter lactaris*, which has been associated with an anti-inflammatory profile through its negative correlation with IL-8)^31^, also increased in abundance. In contrast, *Terrisporobacter mayombei* decreased across dietary groups - potentially beneficial given emerging links to primary ovarian insufficiency^32,33^. Notably, *Intestinibacter bartlettii* decreased; although evidence remains limited, it has been reported as opportunistically enriched in certain conditions (e.g., osteoporosis) and associated with immune-checkpoint-inhibitor–related colitis, while possessing gallate-metabolizing capacity^34–36^.

Taken together, these findings are consistent with selective modulation of commensal taxa following repeated NSSO intake. Given the exploratory nature of this pilot study, they should be considered hypothesis-generating and require confirmation in larger, controlled studies.

Ferrie et al. reported consistent evidence of postoperative dysbiosis following colorectal surgery, characterized by a decrease in beneficial phyla (notably *Firmicutes* and *Actinobacteria*), a relative increase in potentially pathogenic *Proteobacteria*, and reductions in total bacterial load and diversity, indicating impaired microbial resilience and disrupted intestinal homeostasis^6^. Their heterogeneous data, including genus- and species-level analyses, precluded direct species-level comparison. The pattern observed in the present study, however, shows heterogeneous modulation within *Firmicutes* and *Bacteroidota*, accompanied by a consistent decrease of *Actinobacteria*. Under NSSO supplementation, this reorganization coincided with the enrichment of beneficial commensals (*Phocaeicola vulgatus*, *Dysosmobacter welbionis*, *Mediterraneibacter lactaris*,* Blautia schinkii*) and the reduction of taxa with unfavorable associations (*Intestinibacter bartlettii*, *Terrisporobacter mayombei*). Although these findings cannot be directly extrapolated to the perioperative setting, they provide a rationale for investigating whether NSSO supplementation may help modulate microbiome alterations associated with colorectal surgery.

As microbial and host metabolism are closely interconnected, the detection of TQ and its metabolites provides insight into absorption dynamics and systemic exposure following repeated supplementation. In contrast to our previous single-dose study^20^, TQ and/or its metabolite TQ-H₂ were detected in all collected urine samples after repeated administration of TQ-containing NSSO. To our knowledge, literature on the detection of TQ in human samples is scarce^19,20^.

Several limitations must be considered. The open-label design and small sample size increase the risk of bias and limit the generalizability of the findings. The lack of a placebo group precluded complete separation of expectation-related behavioral changes, natural temporal variation in the gut microbiome, and intervention-associated effects. Future studies should therefore employ a blinded, placebo-controlled design. Placebo selection should be made carefully, as oil-based placebos such as flaxseed oil may themselves alter microbial composition and confound attribution to TQ-containing NSSO. Furthermore, although food diaries were reviewed descriptively, no standardized diet, predefined dietary restrictions, controlled meal plans, or formal multivariable dietary adjustment were used. Residual dietary confounding therefore cannot be excluded. The 20-day intervention and short follow-up period do not allow conclusions about durability of microbiome changes or clinical relevance. Finally, urinary TQ/TQ-H₂ detection was based on untargeted GC-MS and should be interpreted qualitatively rather than as pharmacokinetic quantification. Future targeted and validated pharmacokinetic studies are required to evaluate TQ metabolism and excretion dynamics. The detection of TQ and/or TQ-H₂ in urine is compatible with repeated exposure to the study compound and supports the feasibility of urinary monitoring in future studies.

Because repeated measurements were obtained from each participant, the longitudinal study design required statistical methods that accounted for within-participant correlation. Accordingly, differential-abundance analyses were performed using MaAsLin2 with participant ID included as a blocking factor, and PERMANOVA permutations were constrained by participant ID. Nevertheless, given the very small number of participants, exploratory analyses such as LEfSe, Random Forest, and simple between-group comparisons remain susceptible to instability and overestimation of taxon-level differences. Individual taxa should therefore be interpreted as candidate signals for future validation rather than definitive NSSO-associated treatment effects.

## Conclusion

This exploratory pilot study provides preliminary human evidence that repeated intake of TQ-containing NSSO capsules is associated with selective modulation of gut microbial taxa while preserving overall microbiome stability. Qualitative urinary detection of TQ/TQ-H₂ following repeated supplementation supports the feasibility of urinary monitoring in future pharmacokinetic studies. The observed enrichment of several SCFA-producing and potentially anti-inflammatory commensal taxa provides a rationale for further investigating NSSO as a microbiome-modulating strategy in the perioperative setting. These findings require confirmation in larger, adequately powered, blinded, placebo-controlled studies with controlled dietary conditions.

## Supplementary Information

Below is the link to the electronic supplementary material.

Supplementary Material 1

## Data Availability

The datasets analyzed during the current study are available at: https://dataverse.harvard.edu/previewurl.xhtml? token=63ea526d-151b-49fd-9340-753ddf000038.

## Abbreviations

ASV: Amplicon sequence variants
BMI: Body mass index
Ctrl: Control
GC-MS: Gas chromatography mass spectrometry
IBD: inflammatory bowel disease
ICI: immune checkpoint inhibitor
JUH: Jena University Hospital
LC-MS: Liquid chromatography mass spectrometry
NS: Nigella sativa
NSSO: Nigella sativa seed oil
NSSP: Nigella sativa seed polysaccharides
SCFA: Short-chain fatty acids
SDC: Supplementary digital content
TQ: Thymoquinone
Trtm: Treatment
