# Supplementary material for: A prospective randomized controlled pilot study of gut microbiome modulation by Nigella sativa seed oil and thymoquinone metabolism in healthy volunteers: implications for gastrointestinal surgery
Source: Sci Rep. 2026 Sep 15;16:28704. doi: 10.1038/s41598-026-69567-3 (PMC13578717; doi:10.1038/s41598-026-69567-3)
Supplement: Supplementary file 1 [file 41598_2026_69567_MOESM1_ESM.docx]

**Supplementary Digital Content (SDC)**

**SDC 1, Results:** Sample identification and labeling, from patient control groups (C01-C04) and patients under treatment (T01-T04), at -10 (A) and -3 (B) days of the starting time point of administration NSSO, and after 10, 20 and 30 (P) days of treatment.

**SDC 2 Results:** Fecal microbial ASVs coverage of control (red) group and treatment (blue) group reached saturation in all cases.

**SDC 3, Results:** Set of species showed significant increases and decreases (p < 0.05) in Random Forest analyses.
